# P-1517. Assessing the novel combinations against Carbapenem resistant *Acinetobacter baunmannii* (CRAB): ceftazidime-avibactam and ampicillin-sulbactam

**DOI:** 10.1093/ofid/ofae631.1686

**Published:** 2025-01-29

**Authors:** Vyanka Mezcord, Fernando Pasteran, Irene Luu, Miguel Dumas Marucci, Alejandra Corso, Marcelo Tolmasky, Robert A Bonomo, David Paterson, Maria Soledad Ramirez

**Affiliations:** CSUF, Fullerton, California; ANLIS-Malbrán, Buenos Aires, Ciudad Autonoma de Buenos Aires, Argentina; CSUF, Fullerton, California; Malbran, Caba, Buenos Aires, Argentina; Instituto de Enfermedades Infecciosas ANLIS "Dr Carlos G Mabrán", Buenos Aires, Buenos Aires, Argentina; CSUF, Fullerton, California; Case Western Reserve University/ Louis Stokes Cleveland VA Medical Center, Cleveland, OH; The University of Queensland Centre for Clinical Research, Brisbane, Queensland, Australia; California State University Fullerton, Corona, California

## Abstract

**Background:**

Carbapenem-resistant *Acinetobacter baumannii* (CRAB) poses a serious threat to public health, as treatment options are becoming limited. IDSA Guidance has advocated combination therapies to combat CRAB. Sulbactam (SUL), a β-lactamase inhibitor with inherent antibacterial activity against CRAB, is recognized as an important component of combination therapy. Pairing avibactam (AVI) inhibitor with ceftazidime (CAZ), has also been successful in restoring its antibiotic efficacy against many Gram-negative pathogens but has not proven effective against CRAB when used alone. In vitro assays combining SUL and AVI have shown promise against CRAB with different genetic backgrounds. Our aim is to assess the *in vitro* efficacy of combining ampicillin-SUL (AMS) and CAZ-AVI (CZA) against CRAB.

Figure 1

**Figure:**
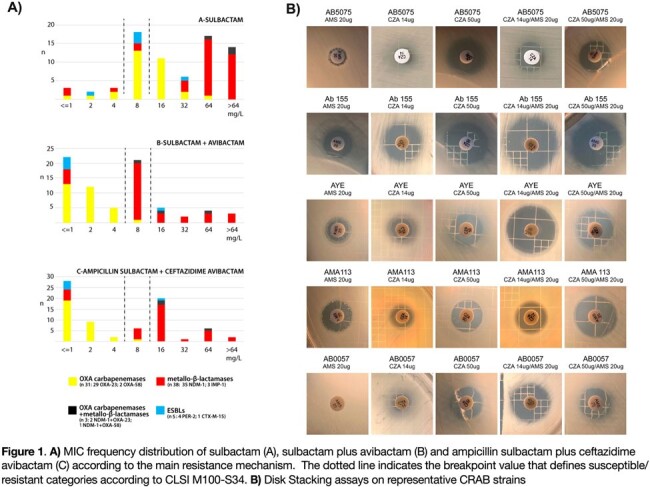

**Methods:**

Three different methodological strategies were used. 89 strains underwent MIC evaluation (agar dilution) against SUL plus AVI, AMS (2:1) plus AVI, and AMS plus CZA, with a constant concentration of AVI (4 mg/L) and CAZ (8 mg/L). Additionally, 30 CRAB strains with different genetic backgrounds were used to assess AMS/CZA (14 μg and 50 μg) synergy by the disk stacking method and MTS synergy.

**Results:**

AVI reduced SUL MIC values to < =4 mg/L in 96.8% of the tested isolates, excluding NDM producers. For MBLs producers, SUL-AVI MIC values less < =4 mg/L were tested in 14.3% strains (Fig. 1A). The inclusion of AMP or CAZ in the SUL-AVI combination did not result in any alterations in MIC values (98% of strains with identical values). Using stacking assay, 59% of isolates showed >3 mm increase in halo diameter for AMS with both CZA disks (Fig. 1B). Additionally, we calculated the FICI by using gradient strips (MTS) obtaining a synergistic or additive effect in 69% and 2% of strains, respectively. Antagonism was only seen in two tested NDM-1 positive isolates.

**Conclusion:**

Our results demonstrated synergy between AMS and CZA in geographically diverse CRAB, including extensively drug-resistant isolates, such as those producing widespread oxacillinases.

Some methods, mainly disk-based, may underestimate the true extent of AMS-CZA synergy. These findings could facilitate the implementation AMS/CZA in clinical settings and provide a potential alternative in regions where the latest approved drugs are not accessible.

**Disclosures:**

**David Paterson, Infectious Diseases Physician**, AMR Action Fund: Board Member|Aurobac: Advisor/Consultant|bioMerieux: Grant/Research Support|bioMerieux: Honoraria|CARB-X: Advisor/Consultant|Entasis: Expert Testimony|Menarini: Honoraria|Merck: Grant/Research Support|Pfizer: Advisor/Consultant|Pfizer: Grant/Research Support|Shionogi: Grant/Research Support|Shionogi: Honoraria

